# Acute generalized exanthematous pustulosis as a manifestation of Kawasaki disease

**DOI:** 10.1007/s12519-021-00450-z

**Published:** 2021-08-18

**Authors:** Wen-Jie Wu, Wen-Qun Zhang, Dao-Zhu Si, Yong-Chun Su, Qin Xie

**Affiliations:** Department of Pediatrics, Chongqing Youyoubaobei Women and Children’s Hospital, No. 999, Jiarong Road, Yubei District, Chongqing, 401147 China

Kawasaki disease (KD) is the leading cause of acquired heart disease in children worldwide. KD patients with an onset of lymph node enlargement had a high incidence of coronary artery complications [1]. It is important to recognize a benign enlargement of lymph nodes and/or various rashes as possibly diagnostic of KD at an early stage.

Acute generalized exanthematous pustulosis (AGEP) is an uncommon but severe cutaneous adverse reaction that is usually caused by medications [2]. It manifests as erythematous pustular eruption and fever, and can be misdiagnosed as KD. Nonetheless, KD with an AGEP-like rash is rare. Here, we report the case of a 5-year-old boy who was diagnosed with KD presenting as an AGEP-like rash.

A previously healthy 5-year-old boy, was admitted with a 6-day history of cervical lymphadenopathy, a 3-day history of rash, and one day of fever without obvious causes. The lymphatic masses on the right side of the neck were tender and did not enlarge progressively. A small polymorphous exanthema on the face and neck developed after eating seafood. One day prior to admission, he developed fever with a body temperature as highest of 39.0 °C and the skin rash gradually spread to the trunk and limbs with pruritus. He was treated with oral antibiotics (amoxicillin with clavulanic acid, 228.5 mg, twice a day) for one day and was in a bad condition, with poor appetite and reduced urine volume. He was allergic to dust mites, seafood, and mushrooms, and his father had a history of rhinitis. The patient’s medical and family histories were otherwise unremarkable.

Physical examination upon admission revealed scattered erythematous and pustular rashes on the trunk and limbs, while the BCG inoculation site scar showed no redness. There was bilateral cervical lymphadenopathy with a palpable enlarged lymph node (3 × 3 cm) in the right anterior cervical triangle on neck palpation. His lips were red and slightly dry, rather than chapped. He also had a mild strawberry tongue and indurative edema of palms and soles, which were alleviated after the fever resolved. No other positive signs were found.

Laboratory findings on admission were: hemoglobin 10.2 g/dL, white blood cells (WBC) 13,900/μL (%neutrophil 0.7, %lymphocyte 0.21, %eosinophil 0.06) and platelets 329,000/μL. C-reactive protein (CRP) was 53 mg/dL and the erythrocyte sedimentation rate (ESR) was 63 mm/hour. Blood biochemistry, urine routine, and blood culture test results were all normal.

The patient was prescribed oral amoxicillin with clavulanic acid (228.5 mg, twice daily) for one day after admission to the hospital. On day 2 of hospitalization, the patient had recurrent fever with a body temperature of 40 °C, and more diffusely distributed pustules on his trunk and extremities (Fig. 1). In this case, sepsis caused by cocci was considered, and he received intravenous cefoperazone sodium (40 mg/kg, q8h) for anti-infection, oral cetirizine (5 mg, QD) for anti-allergy, and other supportive treatments, such as paeonol ointment for antipruritic us, topical use of amikacin, and rehydration.

**Fig. 1:**
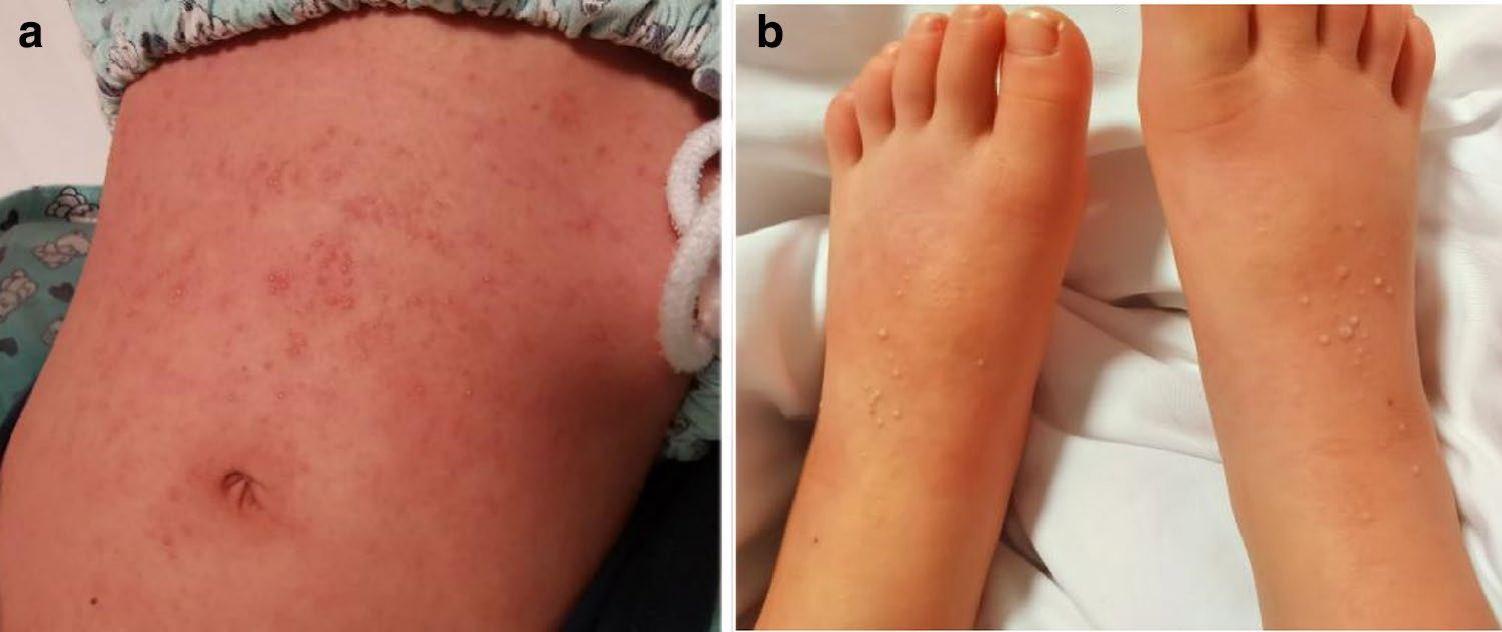
Congestive and pustular rashes on the trunk (a) and limbs (b)

On day 4 post-admission, the enlarged lymph nodes on the neck shrank. The erythematous rashes gradually subsided, while the pustular rashes remained the same. Laboratory results of the pustular puncture fluid were negative. The patient still had fever, bilateral conjunctival hyperemia, dry and cracked lips, and swelling of the hands and feet. Follow-up routine blood test revealed the following: hemoglobin 11.8 g/dL; WBC 13,010/μL; CRP 85.3 mg/dL; and ESR 79 mm/h. Echocardiography was normal, without any signs of obvious coronary artery dilatation. After 5 days of fever and meeting four out of the five criteria for KD, the patient was administered intravenous immune globulin (IVIG) (2 g/kg, once) and aspirin (25 mg/kg, orally, twice daily). The symptoms were effectively alleviated in the following 24 h, and no fever recurred. On the second day after IVIG treatment, his blood routine was normal, and CRP level decreased to 41.8 mg/dL, followed by typical extremity desquamation on day 14 (Fig. 2).

**Fig. 2:**
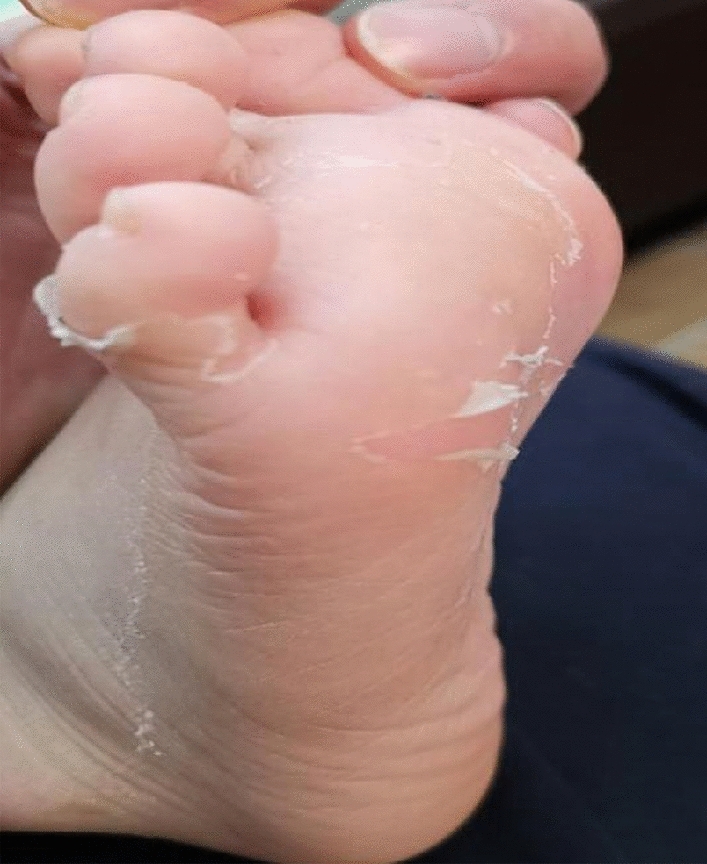
Typical extremity desquamation of the foot

Approximately 90% of KD cases present with a diffuse polymorphous rash that can have a morbilliform, urticarial, micropustular, or other morphology. AGEP-like rash in KD is rare. Only one study reported that the AGEP-like rash in KD was related to the administration of azithromycin [3]. AGEP may be precipitated by medications (most commonly antibiotics) [4], and the cause of AGEP-like rash in this case may have been related to the intake of oral amoxicillin with clavulanic acid based on the early onset of lymphadenitis. AGEP is characterized by the eruption of numerous small sterile pustules on a background of diffuse erythema, typically in the setting of fever. It is characterized by non-follicular pustules, which distinguish it from follicular pustular diseases, such as bacterial folliculitis. Other non-follicular pustular diseases, including pustular psoriasis, can be more difficult to distinguish from AGEP [4]. Pustular psoriasis is slower in onset, and its pustules occur on top of an erythematous base on histopathology, while the majority of intraepidermal pustules are in the upper epidermis and often contiguous with the subcorneal pustules in AGEP. Compared with psoriasis, the rash of AGEP fades quickly without recurrence. Additionally, AGEP has prominent desquamation similar to KD [5]. The difference is that the desquamation of KD starts from the extremities, while AGEP starts from the lesion. Based on the characteristics of desquamation in this case, it was caused by KD.

 The findings of this study may have implications in clinical practice. We should pay attention to the appearance of skin rashes with the use of penicillins and macrolides in KD. The AGEP-like rash of KD may not require any particular treatment after treating the primary disease because it can gradually subside. However, our study has several limitations. The lack of biopsies is a pity for a definite diagnosis of the disease. Immunological tests should be conducted to distinguish from other autoimmune diseases. In conclusion, various rashes including AGEP-like rash may present in KD.

## Data Availability

All data included in this study are available upon request by contact with the corresponding author.
